# Convergent evolution of specialized generalists: Implications for phylogenetic and functional diversity of carabid feeding groups

**DOI:** 10.1002/ece3.6746

**Published:** 2020-10-11

**Authors:** Dennis Baulechner, Frank Jauker, Thomas A. Neubauer, Volkmar Wolters

**Affiliations:** ^1^ Department of Animal Ecology and Systematics Justus Liebig University Giessen Giessen Germany; ^2^ Naturalis Biodiversity Center Leiden The Netherlands

**Keywords:** ecological specialization, evolutionary ecology, functional groups, functional morphology, geometric morphometrics, morphological adaptation

## Abstract

1. Closely related species are often assumed to be functionally similar. Phylogenetic information is thus widely used to infer functional diversity and assembly of communities. In contrast, evolutionary processes generating functional similarity of phylogenetically distinct taxa are rarely addressed in this context.
2. To investigate the impact of convergent evolution on functional diversity (FD) and phylogenetic diversity (PD), we reconstructed the phylogenetic structure of carabid trophic groups. We then analyzed the mandible shapes using geometric morphometrics to link specialization in functional morphology with feeding specialization among herbivores, generalist carnivores, and specialized consumers of Collembola.
3. Our results show that carabid feeding groups are paraphyletic. Herbivory evolved at least twice and specialization to Collembola predation at least three times. Species within feeding groups share a remarkably similar mandible morphology, which evolved convergently. While specialized mandibles of herbivores and collembolan specialists represent an adaptation to their main food source, the particular mandible morphologies do not necessarily reflect the degree of food specialization within feeding groups. Only a few species with a specialized herbivorous mandible may occasionally feed on animals, but the range of specific food resources in generalist carnivore species is large, despite an almost identical mandible shape.
4. Thus, convergent evolution in specialized feeding groups reverses the relationship between PD and functional similarity compared with generalist carnivores. We conclude that phylogenetic relationship is a poor proxy of FD in carabids. Moreover, the inconsistencies between relatedness, morphological adaptation, and ecological function require caution in the characterization of functional groups. Rather than assuming general relationships between PD and FD, we suggest integrating the analysis of evolutionary processes into functional community analyses.

## Graphical abstract

In this study, we demonstrate that in carabids feeding groups and their morphological adaptations to the main food resource evolved multiple times convergently. Therefore, in carabids there is no clear relationship between phylogenetic distance and functional similarity. We further show that morphological specialization does not explain ecological specialization and many species are specialized generalists.

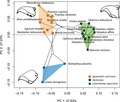

## 1 INTRODUCTION

Convergent evolution is a key issue of evolutionary biology and has important implications for the development of ecological concepts (Harmon, Kolbe, Cheverud, & Losos, 2005). It can shape communities (Losos, 1992; Melville, Harmon, & Losos, 2006; Webb, Ackerly, McPeek, & Donoghue, 2002), adaptive radiations (Muschick, Indermaur, & Salzburger, 2012), and whole ecosystems (Losos, Jackman, Larson, Queiroz, & Rodriguez‐Schettino, 1998; Mahler, Ingram, Revell, & Losos, 2013). Consequently, taking into account convergent evolution as a key mechanism that modulates biodiversity is vital for understanding ecological patterns and processes. For example, species with similar resource use (i.e., feeding groups or guilds) evolved multiple times in different communities, resulting in a remarkable resemblance of trophic patterns (Blondel, 2003). However, the implications of convergent evolution are barely addressed in community ecology. This is surprising, considering the increasing relevance of phylogenetic distance as a proxy for ecological differences in community analyses (Flynn, Mirotchnick, Jain, Palmer, & Naeem, 2011; Kraft et al., 2015). Since it is often assumed that phylogenetic diversity (PD) correlates with functional diversity (FD), PD is often used for assessing community assembly processes and ecological functioning (Cadotte, Cavender‐Bares, Tilman, & Oakley, 2009; Cavender‐Bares, Kozak, Fine, & Kembel, 2009; Srivastava, Cadotte, MacDonald, Marushia, & Mirotchnick, 2012). However, the relationship between PD and FD remains controversial and strongly depends on trait selection and taxonomic scale (Cadotte, Davies, & Peres‐Neto, 2017; Mazel et al., 2018; Tucker, Davies, Cadotte, & Pearse, 2018; Wilcox, Schwartz, & Lowe, 2018).

We focus on two topics that challenge the hypothesis of a general relationship between FD and PD. First, we examine the assumption that this relationship is offset by convergent evolution only in distantly related species (cf. Cadotte et al., 2017). Second, we address the question whether the assignment to functional groups without considering adaptation, specialization, and phylogenetic relationships can bias the conclusions drawn about community structure and assembly. Members of functional groups that are predefined based on coarse taxonomic criteria can have a high overlap in resource use (e.g., guilds *sensu* *stricto*, see Simberloff & Dayan, 1991; Blondel, 2003) or little to no overlap (e.g., generalist carnivores or predators). The degree of resource use overlap thus determines their ecological similarity (or “functionality”) and their reaction to environmental changes.

The use of specific resources requires a specific functional morphology, which should reflect the degree of specialization (Dehling, Jordano, Schaefer, Böhning‐Gaese, & Schleuning, 2016; Ricklefs, 2012). Consequently, convergence in feeding habits requires the independent evolution of morphological adaptations. Our study builds upon the necessity to understand evolutionary processes and morphological adaptations before making general assumptions on the relationship between FD and PD. Carabid beetles are well suited for this purpose, because they comprise several functional groups (here: feeding groups) with different implications for community assembly (Cole et al., 2002; Ribera, Foster, Downie, Mccracken, & Abernethy, 1999; Schirmel, Thiele, Entling, & Buchholz, 2016). For example, herbivorous species can either be specialized on certain seeds or feed on a wide range of seeds (Honek, Martinkova, Saska, & Pekar, 2007), but occasionally even consume insects (Talarico, Giglio, Pizzolotto, & Brandmayr, 2016). Some of these preferences are restricted to specific taxa, for example, certain *Harpalus* and *Amara* species are strongly specialized in seeds (Hengeveld, 1979; Honek et al., 2007). Similarly, while several carnivorous carabids are specialized to prey on collembolans, annelids or mollusks (Kotze et al., 2011), many generalists occasionally also feed on these taxa (Roubinet et al., 2018). Carabid feeding groups thus cover a wide range of specialization levels. In addition, herbivorous species contrast to carnivorous species in having a high overlap in resource use. They can therefore be considered as guilds (Blondel, 2003) exposed to strong interspecific competition, while competition among carnivorous carabids is likely to be low, due to low overlap in resource use.

The analysis of carabid communities is hampered by the fact that the assignment of many species to feeding groups is still based on potentially misleading laboratory observations or a very limited set of field data. This might lead to a serious misinterpretation of the processes driving carabid community assembly. A profound understanding of the relationship between mandible morphology and its adaptive value for exploiting certain food sources could thus be very helpful to overcome this gap in knowledge (Acorn & Ball, 1991; Evans & Forsythe, 1985, 2009; Forsythe, 1983). So far, however, neither the suitability of mandible morphology as a proxy for “feeding groups” nor the associated phylogenetic restrictions have been sufficiently investigated. The same applies to the question of whether mandible morphology reflects the degree of trophic specialization in carabids.

By combining morphological measurements with functional and phylogenetic parameters, we investigate the influence of convergent evolution on the relationship between PD and FD. Specifically, we hypothesize that (a) feeding groups of carabids originate from convergent evolutionary lines, resulting in a high phylogenetic diversity, and (b) mandible morphology evolved convergently as an adaptation to the main food source.

## 2 METHODS

### 2.1 Selection of species and definition of feeding groups

We selected 32 species of carabids, which can be assigned by their main food resource to one of four feeding groups (Table 1): herbivores, generalist carnivores, collembolan specialists, and one genus (*Carabus*) of large carnivores (Fawki, Smerup Bak, & Toft, 2003; Freude, Harde, Lohse, & Klausnitzer, 2004; Hengeveld, 1980; Homburg, Homburg, Schäfer, Schuldt, & Assmann, 2014; Honek, Martinkova, & Jarosik, 2003; Turin, Penev, & Casale, 2003). To analyze the degree of convergent evolution within these groups, we include at least two species with different degrees of specialization to the same feeding group. We follow the nomenclature and taxonomy of Freude et al. (2004).

**TABLE 1:** DNA sequences used for phylogenetic reconstruction in this study

Taxon	Total length	18s	28s	COI	EF1a
*Abax parallelepipedus*	3,875 bp	2,082 (176 in.)	1,146 (128 in.)	647	
*Agonum muelleri*	4,405 bp	2,082 (186 in.)	971 (122 in.)	646 (17 'N')	706 (173 'N', 1 in.)
*Amara aenea*	3,759 bp	2,082 (202 in.)	971 (100 in.)		706 (1 in.)
*Amara apricaria*	3,852 bp	2,082 (196 in.)	1,123 (136 in.)	647	
*Amara aulica*	1,345 bp	476 (115 in.)	222 (20 in.)	647	
*Amara ovata*	647 bp			647	
*Amara plebeja*	647 bp			647	
*Amara similata*	1,343 bp	474 (79 in.)	222 (22 in.)	647	
*Anisodactylus binotatus*	2,951 bp	2,082 (201 in.)	222 (23 in.)	647	
*Bembidion tetracolum*	3,844 bp	2,063 (131 in.)	1,134 (175 in.)	647	
*Calathus fuscipes*	2,082 bp	2,082 (201 in.)			
*Carabus cancellatus*	3,857 bp	2,082 (184 in.)	1,128 (188 in.)	647	
*Carabus nemoralis*	3,906 bp	2,082 (185 in.)	1,177 (187 in.)	647	
*Carabus violaceus*	1,774 bp		1,127 (182 in.)	647	
*Harpalus affinis*	4,380 bp	2,082 (201 in.)	945 (136 in.)	647	706 (2 in.)
*Harpalus latus*	647 bp			647	
*Harpalus luteicornis*	647 bp			647	
*Harpalus rubripes*	1,343 bp	474 (75 in.)	222 (23 in.)	647	
*Harpalus rufipes*	2,798 bp	474 (75 in.)	971 (140 in.)	647	706 (1 in.)
*Leistus ferrugineus*	2,728 bp	2,081 (147 in.)		647	
*Leistus rufomarginatus*	647 bp			647	
*Leistus spinibarbis*	647 bp			647	
*Loricera pilicornis*	3,606 bp	2,041 (165 in.)	222 (34 in.)	637	706 (1 in.)
*Nebria brevicollis*	3,817 bp	2,041 (131 in.)	1,129 (101 in.)	647	
*Noterus clavicornis*	3,837 bp	2,076 (1 '*N*', 160 in.)	1,114 (213 in.)	647	
*Notiophilus palustris*	647 bp			647	
*Notiophilus semiopacus*	3,216 bp	2,082 (172 in.)	1,134 (147 in.)		
*Ophonus ardosiacus*	647 bp			647 (1 'N')	
*Ophonus azureus*	647 bp			647	
*Ophonus laticollis*	2,729 bp	2,082 (201 in.)		647	
*Poecilus cupreus*	1879 bp	1,010 (112 in.)	222 (24 in.)	647	
*Poecilus versicolor*	2,803 bp	996 (111 in.)	1,160 (114 in.)	647	
*Pterostichus melanarius*	4,728 bp	2,082 (187 in.)	1,293 (128 in.)	647	706 (1 in.)
*Synuchus vivalis*	4,406 bp	2,082 (202 in.)	971 (136 in.)	647	706 (15 'N', 1 in.)
*Trachypachus holmbergi*	4,612 bp	2,082 (83 in.)	1,177 (159 in.)	647	706 (1 in.)

Note

Numbers in brackets indicate number of indels (in.).

Abbreviations: bp, base pairs; COI, cytochrome oxidase subunit 1; EF1a, elongation factor 1 alpha.

Herbivorous species were selected from the four genera *Ophonus*, *Anisodactylus*, *Harpalus,* and *Amara* of the two tribes Harpalini and Zabrini, which are known to consist of seed‐feeding carabids (Talarico et al., 2016). We took special care to include species with a different degree of specialization, from granivorous specialists (*Ophonus* spp.) to generalist species (*Harpalus rufipes, Amara similata*), to investigate whether morphological adaptations are reflected in the degree of specialization. We included species with different body sizes (e.g., *Amara aenea* with a max. size of 8.5 mm and *Amara aulica* with 15 mm), since this parameter can constrain the type of seeds that are accessible as a food resource.

Three genera of collembolan specialists (*Loricera*, *Leistus*, *Notiophilus*) are each represented by one species per genus. Members of all three genera are highly specialized and evolved various morphological adaptations to capture collembolans (Bauer, 1981, 1985; Freude et al., 2004; Yin, Cai, Huang, & Li, 2017).

Generalist carnivorous species were selected from several tribes (Pterostichini, Bembidini, and Nebrini). *Nebria brevicollis* (a generalist carnivore, Šerić Jeleska, Franjević, Jeleska, & Symondson, 2014) belongs to the same tribe as *Leistus* (Nebrini). *Poecilus cupreus* is often categorized as an omnivorous species that occasionally consumes seeds, whereas its sister species *P. versicolor* is described as a carnivorous generalist (Homburg et al., 2014). *Pterostichus melanarius* is a generalist predator (McKemey, Symondson, & Glen, 2003), also known as an effective snail predator occasionally consuming seeds (Kulkarni, Dosdall, Spence, & Willenborg, 2015).

*Carabus* species feed on large prey with a preference for snails and earthworms but also insects and other arthropods (Turin et al., 2003). This genus was selected to allow comparison against medium and small generalist carnivores such as *Pterostichus melanarius*, which also feed on annelids and snails.

### 2.2 Phylogenetic reconstruction

Gene sequences for phylogenetic reconstruction were obtained from GenBank for 18s ribosomal RNA, 28s ribosomal RNA, cytochrome oxidase subunit 1 (COI), and elongation factor 1 alpha (EF1a). Sequence length varied across specimens between 647 bp and 4,665 bp (Table 1). COI was available for all but two species. For most genera, at least one species was included with COI, 18s, and 28s. EF1a was available for at least one species per tribe. The beetles *Trachypachus holmbergi* (Trachpachyidae) and *Noterus clavicornis* (Noteridae) served as outgroups. Phylogenetic analyses included two additional species of the genus *Leistus*, in order to increase the phylogenetic resolution in the tribe Nebrini, though specimens for morphological analyses were not available for these species. For *Notiophilus palustris,* only COI sequences are available on GenBank. Therefore, we included *Notiophilus semiopacus* in the phylogenetic analyses and enforced monophyly for these genera. Each gene sequence was aligned using the muscle algorithm in MEGA version 6 (Tamura, Stecher, Peterson, Filipski, & Kumar, 2013). Genes were assembled using the program SequenceMatrix 1.8 (Vaidya, Lohman, & Meier, 2011). We used jModeltest 2.1.5 (Guindon & Gascuel, 2003; Posada, 2008) to determine the best nucleotide substitution model for each gene. For all genes, the general time reversible model (GTR) or a close derivative was determined as the best‐suited explanation for DNA evolution. Therefore, we chose the GTR + Γ + I model for further analyses. Based on this DNA model, we reconstructed ultrametric phylogenetic trees using the software BEAST v1.8 (Drummond, Suchard, Xie, & Rambaut, 2012) based on five independent runs of each 10,000,000 Markov Chain Monte Carlo (MCMC) generations under a strict molecular clock model and based on a Yule speciation process. The MCMC runs were examined using Tracer 1.7.1 (Rambaut, Drummond, Xie, Baele, & Suchard, 2018) to ensure an adequate effective sample size (ESS, >150) and convergence of the MCMC.

### 2.3 Morphological analyses

**FIGURE 1:**
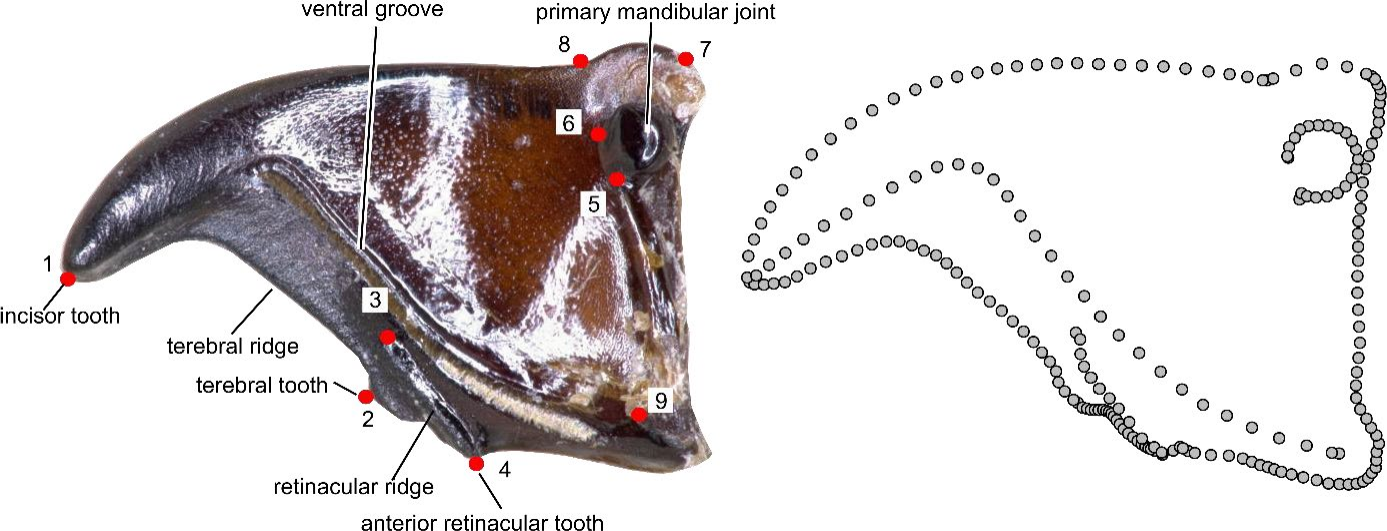
Position of the nine landmarks and 205 semilandmarks along eight curves used to characterize the mandible shape. The example shows the ventral side of the left mandible of *Harpalus rufipes*. The right image displays the position of the SL after sliding

Morphological adaptations of the different feeding groups were assessed based on mandible morphology. Specimens for morphological analysis are part of the collection at the Department of Animal Ecology and Systematics at the JLU and were collected in Hesse in 2011–2012. All specimens, including the mandibles, remain in the collection after dissection. First, the mandibles of three individuals per species were photographed after removing them from the head capsule using a digital microscope (Keyence VHX‐2000; KEYENCE Corp.). Then, nine landmarks (LM) were set on homologous structures occurring on the ventral site of the left mandible of all species by means of the program TPSDig 1.4 (FJ J Rohlf, 2004) (Figure 1). Mandible outline and ridges/grooves were characterized as eight curves using 205 semilandmarks (SL). Each curve was placed between two LM. Nomenclature for the mandibular morphology follows Acorn and Ball (1991). LM 1 was set at the tip of the incisor and LM 2 at the tip of the terebral tooth. The mandible outline in between was connected with a curve of 30 SL. The ventral groove was delimited by LM 3 and LM 4 and connected by a curve of 10 SL. The inferior retinacular ridge was characterized by a curve of 30 SL between LM 1 and LM 9. LM 5 and LM 6 delimited the primary mandibular joint; its outline was characterized by a curve of 20 SL. LM 7 and LM 8 are connected by a curve of five SL. The lateral outline of the mandible was defined by a curve of 30 SL connecting LM 1 and LM 8. The posterior outline of the mandible was defined by a curve of 60 SL between LM 4 and LM 7.

The R package “geomorph” 3.1.0 (Adams, Collyer, Kaliontzopoulou, & Sherratt, 2016; Adams & Otárola‐Castillo, 2013) was used to perform a Generalized Procrustes Analysis (GPA, Rohlf, 1999). Based on the GPA coordinates, an ANOVA was performed to test for statistical differences between the four feeding groups. SL were superimposed based on the minimum bending energy criterion (Bookstein, 1997). Generalized Procrustes coordinates were visualized with a principal component analysis (PCA) including the reconstruction of the phylomorphospace. The mean shape of the three individuals per species was calculated, and the phylogenetic tree was superimposed on the first and the second principal component of the morphospace to construct a phylomorphospace. Outgroups and species from the phylogenetic analyses not represented in the morphological dataset were excluded. To eliminate a potential bias caused by the highly derived mandible of the genus *Carabus*, we conducted a second analysis without this group.

## 3 RESULTS

### 3.1 Phylogenetic relationship within feeding groups

**FIGURE 2:**
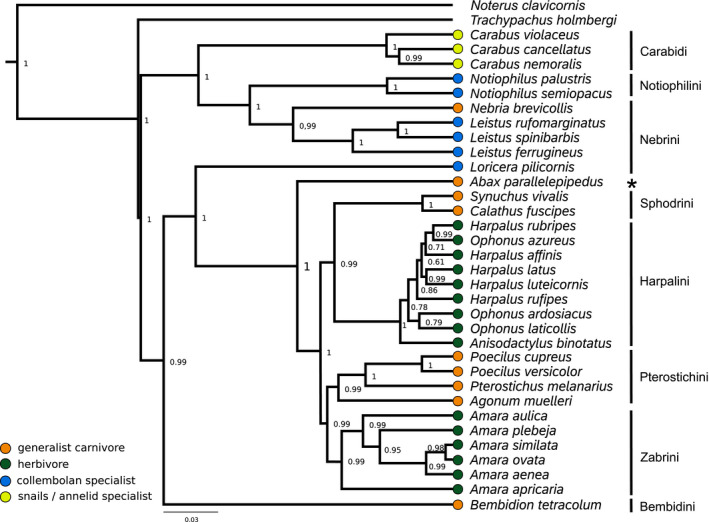
Ultrametric phylogenetic tree based on 18s, 28s, COI, and ef1. Tribes are indicated on the right. Posterior probabilities are given at each node. The asterisks highlight that *Abax parallelepipedus* is generally considered to belong to the tribe Pterostichini

We found strong phylogenetic structure within the feeding groups. According to the reconstructed phylogeny, herbivores, generalist carnivores, and collembolan specialists are not monophyletic groups (Figure 2). Herbivory evolved independently at least twice and specialization to collembolan feeding three times. The two clades of herbivores as well as the three collembolan specialist clades each form monophyletic groups with generalist carnivores. The herbivorous tribe Zabrini and the generalist carnivore tribe Pterostichini are monophyletic. The other herbivorous tribe Harpalini is a sister group to the monophyletic Zabrini‐Pterostichini clade. Due to our limited data and sampling, uncertainties remain as to the phylogenetic position of some groups. However, our results are consistent with other studies (see below) and the phylogenetic tree is well supported. However, our phylogenetic reconstruction placed the generalist carnivore *Abax parallelepipedus* as a sister taxon to all Harpalinae. This position is questionable since the genus is well known to be a member of the Pterostichini (Li, Li, Song, Tang, & Yin, 2020) and might be a result of the very limited data, with only one sequence available for this species. Collembolan specialists belong to three tribes, each forming monophyletic groups with other feeding groups: Loricerini, Nebrini, and Notiophilini. Carabini are opposed to all other taxa. Nebrini include *Leistus* and *Nebria* and are a sister tribe to Carabini and Notiophilini. The herbivorous species of the tribes Zabrini and Harpalini form clades with the generalist carnivorous tribes Pterostichini and Sphodrini, respectively. *Loricera* represents a clade closely related to the Harpalinae (cf. López‐López & Vogler, 2017). The monophyly of Pterostichini and Zabrini and its placement as a sister taxon to Harpalini is consistent with the finding of other studies and supports the convergence of the herbivorous tribes (Ruiz, Jordal, & Serrano, 2009). We can confirm the collembolan specialist genus *Leistus* as a member of the otherwise generalist carnivorous tribe Nebrini (Freude et al., 2004). The collembolan specialists *Loricera* spp. belong in a discrete tribe, which is probably closely related to Harpalinae (compare to López‐López & Vogler, 2017).

### 3.2 Convergent evolution in functional morphology

**FIGURE 3:**
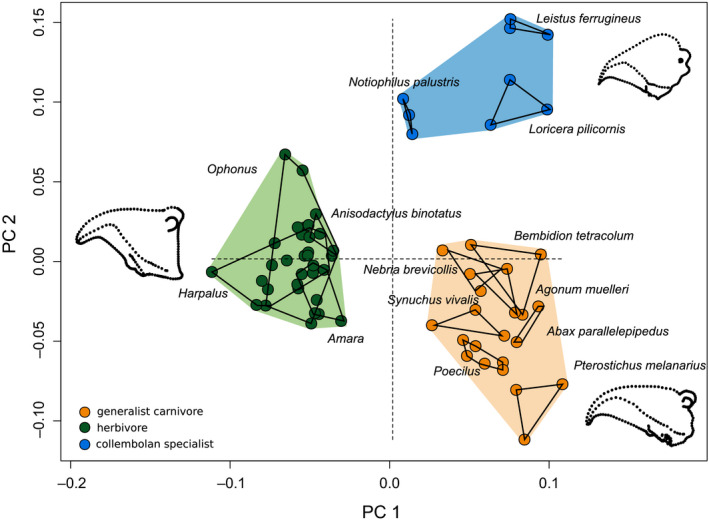
Morphospace of herbivorous (green), generalist carnivorous (orange), and collembolan specialists (blue). PC1: 33%, PC: 25% of the total variance. Each species is represented by three individuals. The three groups differed significantly in mandible shape based on the GPA coordinates (ANOVA *p* < .001)

**FIGURE 4:**
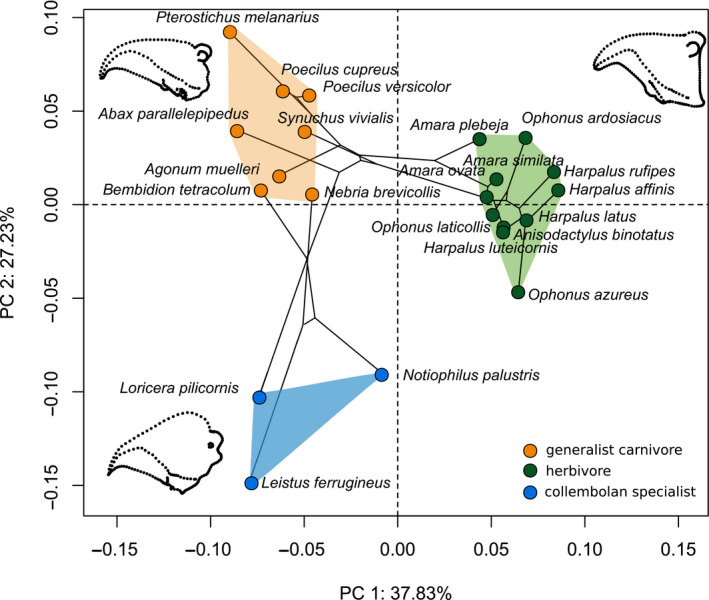
Phylomorphospace of all species excluding the mollusc specialists (*Carabus*), showing the convergent pattern of phylogenetic relationships among feeding groups

The four feeding groups (herbivores, generalist predators, collembolan specialists, and species of the genus *Carabus*) can be identified and grouped according to their mandible morphology (ANOVA *p* < .001, Figure 3). Mollusk–annelid specialists are separated from all other morphotypes (see Appendix S1). Since this strong effect masked obvious differences among other groups, further analyses were confined to the remaining feeding groups (Figure 3). The first two PCs explain 57.4% of the total variance (PC 1:32.9%, PC 2:24.5%). The phylomorphospace analysis based on the mean PC scores of the three individuals per species revealed no corresponding phylogenetic clustering but indicate convergence of morphotypes (Figure 4). PC 1 separates herbivorous species from all other feeding groups (Figures 3 and 4). Adaptation to herbivory obviously selects for very stout mandibles with a rectangular proximal base and much bigger primary mandibular joints (Figure 4) compared with the other two groups, likely to enable the shredding of tough plant material and seeds. The terebral tooth, retinacular tooth, retinacular ridge, and the ventral groove form wide ridges and broad structures probably as an adaptation toward seed consumption (cf. Figure 2, Acorn & Ball, 1991). The phylomorphospace (Figure 4) highlights the strong selection pressure favouring the convergent evolution of this specific mandible shape as an adaptation to herbivory. Herbivorous species show a greater within‐ than between‐species variation, so their mandible morphology cannot be assigned to a specific species. Conversely, at the species or at least the genus level, the collembolan specialists and most generalist carnivores form discrete groups in the morphospace (Figure 3). The tooth structure in the posterior area of the carnivore mandible is more delicate compared with the herbivore mandibles. Moreover, the incisor of carnivorous species is more strongly bent and has a more articulated cutting edge. PC 2 separates collembolan specialists from generalist carnivores (Figures 3 and 4). The most prominent feature separating collembolan specialists from the other groups is the position and much smaller size of the primary mandibular joint, which connects the mandible to the head capsule (Figures 1 and 3). Further, the cutting edge formed by the ventral groove of the pointy and delicate incisor of collembolan specialists is hardly visible. Herbivores and collembolan specialists each evolved morphologically highly specialized mandibles, clearly separating feeding groups. The stout mandibles of both tribes show many morphological adaptations toward seed predation (Acorn & Ball, 1991). Additionally to the findings of Acorn and Ball (1991), we point out the enlargement of the mandibular joint and the more ridge‐like structure of the posterior teeth as an adaptation toward seed consumption. The latter probably serves a more grinding function than the more delicate structures with many single teeth in generalist carnivores.

## 4 DISCUSSION

Convergent evolution of carabids resulted in phylogenetically diverse feeding groups with remarkably similar adaptations in mandible morphology. Our study demonstrates that mandible shape is a good predictor for the primary food source in specialized feeding groups such as herbivores and collembolan specialists. It also shows that there is no general relationship between functional similarity and phylogenetic diversity (PD). The relationship is even reversed between specialist feeding groups such as herbivores and collembolan specialists compared with generalist carnivores.

Convergent evolution resulted in high PD in the herbivorous and collembolan specialist feeding groups. Generalist carnivores are comprised of multiple unrelated groups and, accordingly, also phylogenetically highly diverse. Thus, PD per se is a poor predictor of functional diversity (FD) in carabid communities and might not be affected at all by community responses of feeding groups to environmental change (Baulechner, Diekötter, Wolters, & Jauker, 2019). We found evidence that convergence can cause a discrepancy between phylogenetic and functional divergence not just at broad taxonomic scales (Cadotte et al., 2017) but even within a family at the genus level. Therefore, in line with other recent studies (Mazel et al., 2018), we find that PD does not reliably capture FD and should not be used alone to assess community assembly or functionality. In particular, assembly processes such as competition should not be derived from single measures such as PD.

Our results indicate strong selection pressures for the mandible shape to access specific food resources. However, mandible specialization does not necessarily reflect the degree of specialization and the overlap in resource use. There are many specialists that exclusively feed on seeds, such as the genus *Ophonus* or some *Amara* species, which are even specialized on the seeds of specific plant species (Honek et al., 2003). Despite the overall similarity in specialized morphology, these groups contain many species with a generalist diet. *Harpalus rufipes,* for example, preys on a variety of seeds but also on slugs, spiders, and insects. Moreover, prey spectrum and the degree of specialization vary across seasons (El‐Danasoury, Cerecedo, Córdoba, & Iglesias‐Piñeiro, 2017; Loughridge & Luff, 1983; Roubinet et al., 2018). *Amara similata* is known to feed on aphids, but granivory plays a vital role in its diet (Jorgensen & Toft, 1997). Yet mandible morphology does not reflect the differences in the degree of specialization. A comparable inconsistency in phenotypic and ecological specialization, which is termed Liem's paradox, has also been documented for other taxa such as cichlid fish (Binning, Chapman, & Cosandey‐Godin, 2009; Liem, 1980). Morphological specialization of generalist species might be a competitive advantage when other food sources are scarce (Robinson & Wilson, 1998). The morphological specializations in herbivorous species can be sustained via natural selection as an adaptation as “specialized generalists” given that the access to this resource is ecologically and evolutionarily crucial. Accordingly, the herbivore mandible shape is a good indicator for seeds as a primary food source and supports the classification, despite the occasional carnivorous behavior of some species.

On the other hand, many generalist carnivores occasionally feed on seeds or collembolans and are therefore often considered omnivorous in community analyses. For example, *Poecilus cupreus* is widely considered omnivorous and *P. versicolor* carnivorous (Bargmann, Heegaard, Hatteland, Chipperfield, & Grytnes, 2016; Homburg et al., 2014), although *P. cupreus* may eat seeds under starvation in laboratory conditions (own unpubl. observation). A functional distinction between the two species, based solely on single observations (Homburg et al., 2014; Lindroth, 1986), may bias analytical results regarding community assembly. As we could not find any adaptation toward seed consumption in the mandible morphology of carnivorous generalists, and considering the strong adaptation to seed predation we found in herbivorous species, the ecological relevance of seed consumption in generalist carnivores is questionable. Since mandibles of generalist carnivores are not robust enough to handle seeds as a primary food source, they would get severely battered over time (Wallin, 1988). In addition, there are no studies providing evidence that generalist carnivores rely on seeds under natural conditions or have any influence on plant occurrence by seed predation. Jointly categorizing carnivores that occasionally ingest seeds and highly adapted herbivores that regularly consume large amounts of seeds as “omnivores” results in an inconsistent feeding group.

Thus, feeding groups of carabids are too inconsistent to be useful in the analysis of ecological communities. Overlap in resource use is high among herbivores but very low (or even nonexistent) among generalist carnivores. Moreover, herbivores might also react differently to different ecological conditions depending on their degree of specialization. Despite both being herbivorous, for example, Zabrini and Harpalini strongly differ in the types of seeds ingested, due to strong differences in body size (Honek et al., 2007). We therefore suggest avoiding the term “guild” to classify feeding groups in carabids, a term that has often been used inconsistently in the past anyway (Simberloff & Dayan, 1991). This is supported by the poor evidence of competition for food sources (Kotze et al., 2011) in carabids and the fact that a generalist carnivorous species might occupy different trophic niches (Zalewski et al., 2014). Only collembolan specialists and some herbivores might form guilds in the strict sense, because of the strong similarity in their food spectra. This is reflected in their highly specialized mandibles and the associated high degree of different morphological adaptations, such as setae traps to catch collembolans (Bauer, 1985; Yin et al., 2017).

The high diversity in resource use also becomes evident through the different number of species occurring in the individual feeding groups. In fact, the degree of specialization and species richness are negatively related to each other. With more than 350 species, generalist carnivores constitute the most species‐rich feeding group in central Europe. The herbivorous tribes encompass approximately 55 species and occur in almost every central European habitat. Conversely, the diversity of collembolan specialists is low (Freude et al., 2004).

## 5 CONCLUSION

The repeated convergent evolution of feeding groups obscures a clear relationship between relatedness and ecological functioning regarding the food resource. Equally, the range of specialist species to generalists cannot be explained by phylogenetic relation or morphological adaptation but through convergent evolution. Specialization and generalism can be driven by competition and can have evolutionary (niche evolution) and ecological (e.g., competitive exclusion) consequences (Poisot, Bever, Nemri, Thrall, & Hochberg, 2011). Therefore, community assembly processes such as competitive exclusion cannot be inferred by phylogenetic pattern alone. The same accounts for other assembly processes such as environmental filters, which might select closely related or convergently evolved distantly related species.

## CONFLICT OF INTEREST

The authors report no competing interest.

## AUTHOR CONTRIBUTION

**Dennis Baulechner:** Conceptualization (lead); Data curation (lead); Formal analysis (lead); Investigation (lead); Methodology (lead); Writing‐original draft (lead). **Frank Jauker:** Conceptualization (supporting); Writing‐original draft (supporting); Writing‐review & editing (supporting). **Thomas A. Neubauer:** Formal analysis (supporting); Methodology (supporting); Validation (supporting); Writing‐review & editing (supporting). **Volkmar Wolters:** Conceptualization (supporting); Writing‐original draft (supporting); Writing‐review & editing (supporting).

## Supporting information

Appendix S1‐S2

## Data Availability

Mandible landmarks and genebank accession numbers for phylogenetic reconstructions are provided in the Supporting Information.
