# Supplementary material for: Convergent evolution of specialized generalists: Implications for phylogenetic and functional diversity of carabid feeding groups
Source: Ecol Evol. 2020 Oct 11;10(20):11100–10. doi: 10.1002/ece3.6746 (PMC7593144; doi:10.1002/ece3.6746)
Supplement: Supplementary file 1 — Appendix S1‐S2 [file ECE3-10-11100-s001.zip › ece36746-sup-0001-AppendixS1.docx]

Appendix 1.

Table 1.: Genbank Accession codes of the sequences used for the analyses.

The clear separation of Carabid species from all remaining groups masked obvious differences among other groups. Therefore, further analyses were confined to the remaining feeding groups.
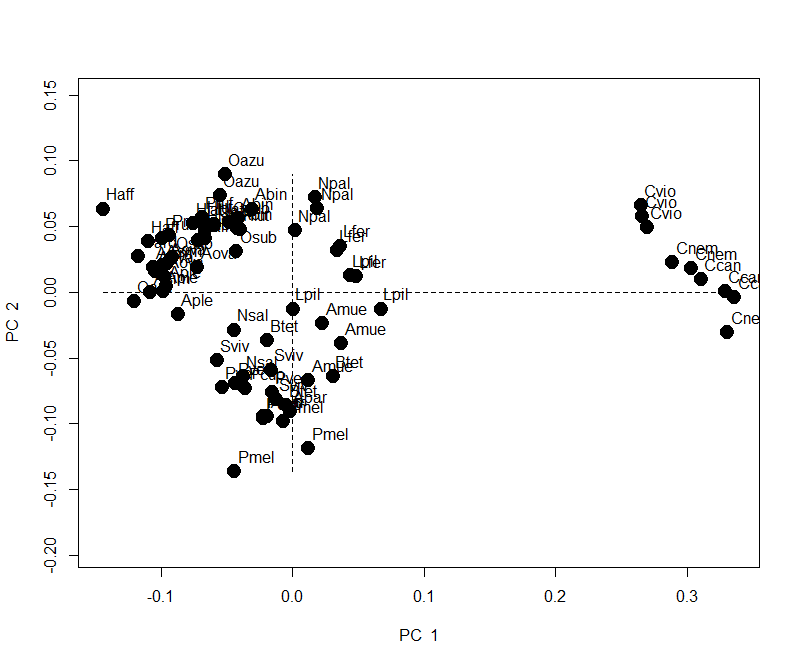


Figure 1: Plot of the tangent space of the mandible of all investigated species. The genus Carabus is clearly separate from all other species on PC 1.
